# Artificial Intelligence in Orthopaedics: Clinical Performance, Limitations, and Translational Readiness—A Review

**DOI:** 10.3390/jcm15051751

**Published:** 2026-02-25

**Authors:** Wojciech Michał Glinkowski, Antonina Spalińska, Agnieszka Wołk, Krzysztof Wołk

**Affiliations:** 1 Center of Excellence “TeleOrto” for Telediagnostics and Treatment of Disorders and Injuries of the Locomotor System, Department of Medical Informatics and Telemedicine, Medical University of Warsaw, 02-091 Warsaw, Poland; 2 The Polish Telemedicine and eHealth Society, Targowa 39A/5, 03-728 Warsaw, Poland; agnieszka.wolk@telemedycyna.org; 3 Department of Biostatistics and Research Methodology, Faculty of Medicine, Cardinal Stefan Wyszyński University, 01-938 Warsaw, Poland

**Keywords:** artificial intelligence, orthopaedic imaging, arthroplasty, osteoarthritis, predictive analytics, rehabilitation intelligence, patient-reported outcomes, clinical implementation

## Abstract

**Background/Objectives:** Musculoskeletal disorders and their surgical treatment significantly affect global disability, healthcare utilization, and costs. Artificial intelligence (AI) is a key enabler of data-driven musculoskeletal care. Their applications include diagnostic imaging, surgical planning, risk prediction, rehabilitation, and digital health ecosystems. This narrative review synthesizes current evidence on the use of AI in orthopaedics and musculoskeletal care across five areas: diagnostic imaging, surgical planning and intraoperative augmentation, predictive analytics and patient-reported outcomes, rehabilitation intelligence and teleorthopaedics, and system-level management. An additional task is to identify translational gaps and priorities for safe, ethical, and equitable implementation of AI. **Methods:** A structured narrative review was conducted using targeted searches in PubMed, Scopus, and Web of Science supplemented by semantic and citation-based explorations in Semantic Scholar, OpenAlex, and Google Scholar. The main search period was January 2019 to December 2025. The retrieved peer-reviewed articles were analyzed for clinical relevance to human musculoskeletal care, quantitative outcomes, and the translational implications of the results. From the broader pool of eligible publications, 40 clinically relevant studies were selected for detailed synthesis covering imaging, surgical planning, predictive modeling, rehabilitation, and system-level applications. Owing to the significant heterogeneity in the model architectures, datasets, and endpoints, the results were organized into five predefined thematic areas. **Results:** The most mature evidence is for AI-assisted detection of bone fractures on radiographs, identification of implants, and use of sizing templates in preoperative planning for arthroplasty, where deep learning systems have achieved expert-level diagnostic performance (e.g., fracture detection sensitivity of approximately 90% and specificity of approximately 92% and implant identification accuracy of 97–99%) and improved the accuracy of preoperative planning compared to conventional templating. AI-based planning increases the likelihood of reducing intraoperative corrections, shortening surgery time, reducing blood loss, and improving the final functional outcomes. Predictive models can support the stratification of risk for complications, rehospitalizations, and patient-reported outcomes, although external validation remains limited and is often single-center at this stage of research. Emerging applications in rehabilitation and teleorthopaedics, including sensor-based monitoring and learning systems integrated with Patient-Reported Outcome Measures (PROMs), are conceptually promising, but are mainly limited to feasibility or pilot studies. **Conclusions:** AI is beginning to influence musculoskeletal care, moving beyond pattern recognition toward integrated, patient-centered decision support throughout the perioperative and rehabilitation periods. Its widespread use remains constrained by limited multicenter validation, dataset bias, algorithmic opacity, and immature regulatory and governance frameworks. Future work should prioritize prospective multicenter impact studies, repeatable revalidation of local models, integration of PROM and teleorthopedic data with health learning systems, and adaptation to changing regulatory requirements to enable safe, ethical, effective, and equitable implementation in routine orthopedic practice.

## 1. Introduction

Musculoskeletal disorders and injuries are among the leading causes of pain and disability worldwide, leading to a significant demand for imaging services for urgent and elective surgeries and rehabilitation, thereby placing a considerable burden on healthcare systems [1,2,3,4,5]. Clinical decision-making in this field involves comprehensive radiological assessment, determination of surgical indications, postoperative observation, and long-term functional monitoring. The increased interest in data-driven tools for the digital transformation process stems from their potential to improve the consistency, scalability, and decision support throughout the orthopedic care process [6,7,8,9]. Orthopaedics is a unique field because of the importance of visual data (X-rays, CT scans, and MRIs) and biomechanical measurements. This makes it ideal for computer vision applications that use deep learning models to automatically extract features from pixel data, unlike traditional machine learning methods that require manual feature definition.

It has been observed that artificial intelligence (AI), especially deep learning and other machine learning-based methods, is advancing rapidly from theoretical concepts to practical applications in orthopaedics [8,10,11,12,13,14,15,16,17,18,19,20]. It has already been demonstrated that convolutional neural networks can achieve high accuracy in fracture detection, comparable to that of musculoskeletal radiology specialists, with overall sensitivities and specificities of approximately 90% and 92%, respectively [21,22,23,24,25]. Systems supporting elective orthopedic surgery with implant placement achieve 97–99% accuracy and can significantly speed up the planning of complex revision arthroplasty procedures [26,27,28]. AI-assisted arthroplasty planning improves implant sizing compared to conventional two-dimensional templating (manual or digital “overlaying” of implant templates onto standard two-dimensional radiographs to estimate the size and positioning of components before surgery) [29,30,31]. Compared with traditional methods, this results in a more efficient procedure, shorter operating times, reduced blood loss during surgery, and fewer intraoperative corrections in hip and knee arthroplasty [32,33,34,35]. In addition to imaging and templating, AI-based predictive models are increasingly used to assess the risks of venous thromboembolism, dislocation, readmission, and patient-reported satisfaction [36,37,38]. Integrating imaging, clinical data, signals from wearable sensors, and patient-reported outcomes (PROMs) to support personalized treatment pathways creates opportunities for multimodal structures [39,40]. The simultaneous use of teleorthopaedics and AI-assisted rehabilitation tools aims to extend specialized care to patients’ homes through remote monitoring, automatic motion analysis, and adaptive exercise guidance [41,42,43]. Despite this promising progress, clinical implementation of AI-based systems remains inconsistent. Many such systems are trained on data from a single center or demographically homogeneous datasets [44,45,46,47], lack robust multicenter external validation, exhibit algorithmic opacity, undermine clinician trust, and raise concerns regarding fairness and accountability. Workflow integration with picture archiving and communication systems (PACS), electronic health records, and surgical planning platforms is often incomplete [44,46]. Regulatory and ethical frameworks for adaptive data-driven tools are still under development. Existing reviews typically focus on single domains, such as fracture detection or arthroplasty planning, and rarely combine diagnostic, surgical, prognostic, rehabilitation, and systemic perspectives into a unified picture of orthopedic healthcare [22,23,48,49,50,51,52].

### Technical Foundations of AI in Orthopaedics

To enhance the understanding, it is essential to outline the core AI techniques that are relevant to this field. Deep learning (DL), a subset of ML, uses neural networks with multiple layers to learn hierarchical features from data and has driven major advances in medical image analysis [53]. In imaging, convolutional neural networks (CNNs) are predominant for tasks such as fracture detection because they excel in processing grid-like data such as radiographs [54]. For the predictive analytics of tabular clinical data, models such as random forests or gradient boosting machines (e.g., XGBoost) are employed for risk stratification and effectively handle structured inputs [55]. These techniques typically require large labeled datasets for training, which are often augmented via transfer learning to mitigate data scarcity in orthopaedics.

In addition to these established model families, emerging architectures such as vision transformers (ViT) and hybrid CNN–transformer pipelines are increasingly employed for image classification and segmentation, particularly when long-range contextual relationships or multiview integration are crucial [56,57,58,59]. In practice, the selection of architecture involves trade-offs between data requirements, robustness, and interpretability [60,61]. CNNs typically excel in tasks characterized by high contrast and well-defined geometry (e.g., implant identification) [62,63], whereas osteoarthritis grading often entails subtle, spatially heterogeneous changes that benefit from precisely constrained regions of interest, multiscale feature extraction, and rigorous external validation [64,65,66]. For structured clinical datasets, gradient-boosting models frequently remain competitive owing to their efficacy with tabular inputs, superior calibration of uncertainty, and more accessible feature attribution than deep neural networks [67]. However, they may underperform when the predictive signal is embedded in high-dimensional imaging or multimodal data [67,68].

This structured narrative review synthesizes current evidence on AI in orthopedic and musculoskeletal practice across diagnostic imaging, surgical planning and intraoperative augmentation, predictive analysis and precision stratification, rehabilitation intelligence and teleorthopaedics, and system-level management using digital ecosystems. This study aimed to characterize the technological maturity and clinical utility of current applications, identify key translational gaps, and outline a research and implementation agenda to support the safe, ethical, equitable, and patient-centered implementation of AI in modern musculoskeletal healthcare.

## 2. Materials and Methods

### 2.1. Study Design

This study was a structured narrative review that combined a targeted literature database search with expert interpretation in the clinical and methodological domains. The synthesis was based on two complementary streams of evidence: (1) a targeted set of clinical studies on AI in orthopedic imaging, surgical planning, and predictive modeling, and (2) a thematic analysis of emerging areas, including PROM–AI integration, teleorthopaedics, and digital rehabilitation.

### 2.2. Search Strategy

This expert review synthesizes current evidence on the clinical applications of AI in orthopaedics and the broad treatment of musculoskeletal disorders. The search covered the period from January 2019 to December 2025, during which deep learning techniques matured into clinically applicable tools and the first regulatory approval for AI-based imaging systems was obtained [69]. This timeframe was deliberately chosen to include studies that reflected actual translational readiness rather than early concept verification.

A comprehensive literature search was conducted using PubMed, Scopus, and Web of Science databases. To increase coverage and minimize database bias, targeted semantic- and citation-based searches were performed using Semantic Scholar, OpenAlex, and Google Scholar databases. The search strategies combined controlled vocabulary and text terms related to AI, machine learning, deep learning, orthopaedics, musculoskeletal disorders, fracture detection, osteoarthritis, arthroplasty, spine surgery, outcome prediction, patient-reported outcome measures (PROMs), PROMIS, telemedicine, teleorthopaedics, and digital rehabilitation.

### 2.3. Eligibility Criteria and Study Selection

Eligible articles comprised original research evaluating AI-based tools or models with direct applicability to clinical practice in orthopaedics, including diagnostic imaging, preoperative planning, risk prediction, rehabilitation, and outcome assessment. Both retrospective and prospective clinical studies were deemed eligible for inclusion. Exclusions were made for conference abstracts, editorials, articles focused solely on algorithm development without clinical validation, and publications in languages other than English. Titles and abstracts were screened to exclude studies outside the scope of clinical orthopaedics and musculoskeletal patient care. Animal studies, investigations focused purely on engineering without a clinical framework, studies on robotics or navigation systems lacking elements pertinent to clinical decision-making, and image-processing studies without clinical validation were excluded from the review. Some included articles, particularly review articles, were used to inform contextual synthesis but were not subjected to primary quantitative data extraction.

The main reasons for full-text exclusion after eligibility assessment are summarised in Supplementary Table S1. The categorisation in Supplementary Table S1 reflects the dominant reason for excluding each full-text article; when multiple exclusion criteria were applied, studies were assigned to the most clinically relevant category.

The reference lists of the included articles and recent high-quality reviews were manually screened to identify additional relevant studies not captured by the electronic search. Full-text articles were assessed for conceptual relevance, availability of quantitative performance data, and translational implications of the results. Rather than applying rigid eligibility thresholds, a priority-based approach was adopted that emphasized clinical relevance. Preference was given to studies reporting diagnostic performance metrics (e.g., sensitivity, specificity, area under the curve), benefits in surgical planning compared with conventional methods, predictive differentiation of outcomes or complications, measurable impact on clinical workflow, or conceptually robust frameworks for rehabilitation and precision medicine. The included studies demonstrated broad geographical representation, with research conducted in the United States, Europe, and Asia (including Sweden, Austria, France, The Netherlands, China, Thailand, India, Japan, and Singapore). Sample sizes varied widely, ranging from small proof-of-concept cohorts (*n* = 12) to large-scale systematic reviews encompassing more than one million implants. Study quality was assessed using the modified Methodological Index for Non-Randomized Studies (MINORS) or the Newcastle–Ottawa Scale for non-randomized studies [70]. These instruments were applied qualitatively to contextualize the strength of evidence and recurrent sources of bias across study designs rather than to generate pooled scores or exclude studies based on numerical thresholds. From the pool of eligible publications, 40 studies were selected as the evidence base for a detailed narrative synthesis.

### 2.4. Data Extraction and Synthesis

Each selected study was analyzed in terms of the area of AI application (diagnostic imaging, surgical planning, predictive analytics, rehabilitation, or system-level), orthopedic specialty, anatomical area, basic AI methodologies, data modality, study design and validation level, quantitative performance metrics, and patient-reported clinical outcomes or workflow impact. A structured organization of predefined data elements was used. All collected information was verified by the authors and placed in context to ensure the clinical accuracy and interpretability of the data.

In this study, it was not possible to combine quantitative data due to significant heterogeneity in model architectures, datasets, and endpoints. The results were synthesized in narrative form, with greater interpretive weight given to empirically verified clinical trials and feasibility studies than to other studies. Simultaneously, new and rapidly evolving ideas, such as integrating patient-reported outcomes (PROMs) with AI, developing tele-orthopedic teaching systems, modeling biological responses, and using digital twin technologies, have been considered preliminary or exploratory. In other words, these concepts were treated as areas that generated hypotheses that required further research and validation before being translated into clinical practice.

### 2.5. Organization and Evaluation of Evidence

**Figure 1:**
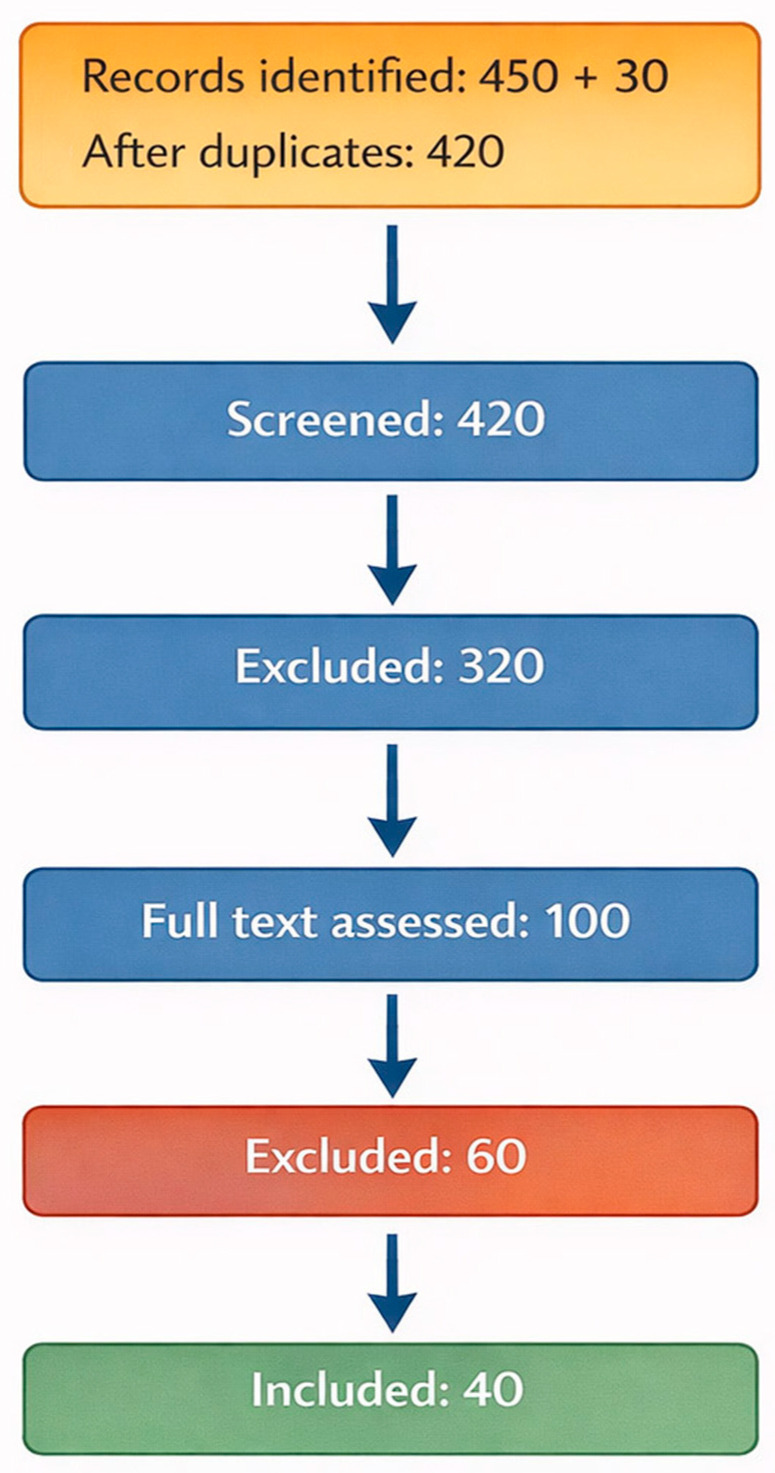
PRISMA-inspired flow diagram. The diagram summarizes the identification, screening, eligibility assessment, and inclusion of studies in the narrative synthesis: 450 records were identified through database searches and 30 via reference lists; after the removal of duplicates, 420 records were screened by title and abstract, 100 full-text articles were assessed for eligibility, and 40 studies were included in the final synthesis.

The results were categorized into five thematic areas to facilitate coherent synthesis and identify translational gaps: (1) AI-based diagnostic imaging; (2) surgical planning and intraoperative augmentation; (3) predictive analytics and precision stratification; (4) rehabilitation intelligence and remote monitoring; and (5) system-level management, ethics, and future research directions. The study identification, selection, and inclusion processes are summarized in a PRISMA-inspired flow diagram (Figure 1).

No formal quantitative grading of evidence (e.g., GRADE or AMSTAR) was conducted because the primary objective was comparative clinical interpretation rather than effect-size aggregation. Instead, studies were qualitatively assessed based on the strength of their clinical signals, with a clear distinction between empirical outcome studies, feasibility studies supported by empirical results, and conceptual or framework-based contributions to the literature.

A formal meta-analysis was not performed due to substantial heterogeneity across study designs, data sources, AI architectures, outcome measures, and validation strategies. Consequently, the results were synthesized narratively, with an emphasis on clinical performance indicators, validation levels, generalizability, and implementation readiness.

The main reasons for excluding full texts are summarized in Supplementary Table S1.

Because this work is a structured narrative review, the flow diagram is intended to increase transparency in the identification and selection of studies. However, the review did not claim formal PRISMA compliance and did not provide a quantitative meta-analytic synthesis.

Grammarly and PaperPal were used for grammatical and stylistic corrections.

## 3. Results

### 3.1. Evidence-Based Review

A narrative synthesis of 40 studies was conducted to reflect the current scope, maturity, and complexity of artificial intelligence applications in orthopaedics. Qualitative assessment using the modified Methodological Index for Non-Randomized Studies (MINORS) and Newcastle–Ottawa Scale revealed that the overall methodological quality of the included studies was predominantly moderate. Approximately two-thirds of the studies met the basic criteria for internal validity, whereas only seven of 40 (17.5%) satisfied the key criteria for a prospective design or external multicenter validation. A qualitative overview of the recurrent methodological strengths and limitations identified using these tools is presented in Supplementary Table S2.

Regarding study design, seven studies (17.5%) utilized prospective or multicenter validation approaches, including randomized controlled trials and clinical trials. The remaining evidence was predominantly composed of retrospective, single-center studies, and development or internal validation cohorts. Common methodological limitations included small sample sizes, lack of prospective validation, incomplete reporting of model calibration, uncertainty, and failure modes.

The evidence base comprised systematic reviews and meta-analyses, cohort and validation studies, randomized controlled trials, and clinically oriented feasibility studies. As summarized in Table 1, most studies have focused on diagnostic imaging and surgical planning, with a small but growing body of evidence on predictive modeling, rehabilitation, and system-level applications.

### 3.2. Diagnostic Imaging Using AI

The most mature and clinically relevant evidence concerns the use of AI in musculoskeletal imaging, particularly in the radiographic detection of fractures [25,47,49,51,52,54,55], implant identification [56,57,58], and osteoarthritis classification [105,106]. In many studies, deep learning systems have achieved a fracture detection sensitivity of approximately 90% and specificity of approximately 92%, with area under the curve values approaching 0.97, matching those of experienced clinicians, outperforming less experienced specialists, and providing millisecond-level inference times [107].

The differences in results across individual studies can be attributed to several factors, including differences in image acquisition and preprocessing protocols, case diversity, and disease prevalence, the method used to determine objective truth (whether through expert consensus or clinical observation/CT), and discrepancies in external validation strategies.

AI algorithms for orthopaedic implant identification demonstrate even higher performance, with reported accuracies of 97–99%, and can substantially accelerate the planning of complex revision arthroplasty procedures [73,108]. In contrast, AI-based classification of knee osteoarthritis, which is clinically useful and associated with improved inter-observer agreement compared with manual assessment, generally achieves lower performance, with reported accuracies of up to 93% [105,109,110]. However, deep learning models must be trained in the selected areas of interest. Otherwise, they lack precision and struggle to identify significant changes that are indicative of osteoarthritis [111]. This discrepancy seems to stem not from the sophistication of the algorithms but from the fundamental differences in image characteristics and task structure. Orthopedic implants are characterized by smooth, well-defined edges, high signal contrast, and a limited set of repetitive shapes, which closely match the strengths of convolutional neural networks in detecting high-contrast global patterns [112]. In addition, image preprocessing strategies, such as contrast enhancement, may further improve recognition in challenging scenarios, including cases of cement adhering to implant surfaces [113]. In contrast, osteoarthritic changes are spatially heterogeneous, gradually evolving, and lack clear geometric boundaries, rendering reliable classification inherently more complex and sensitive to disease stage and imaging variability [114]. Notably, many imaging models continue to rely on single-center datasets and have limited external multicenter validation, further limiting their generalizability.

### 3.3. Surgical Planning and Intraoperative Augmentation

Evidence confirms the significant benefits of AI-assisted planning for hip and knee arthroplasties and their early application in spinal deformity surgeries. In total hip arthroplasty, AI-based preoperative templates and segmentation improved component sizing and positioning compared to manual planning, increasing the acetabular component fit from approximately 30 to 57% to 66% to 90% and enabling the prediction of tibial and femoral components in total knee arthroplasty with an accuracy of approximately 92.9% compared to approximately 45% for manual planning [31,81,115]. AI-assisted planning is associated with shorter operating times (approximately 12 min), reduced blood loss during surgery (approximately 50 mL), and a 40% reduction in intraoperative revisions, with a slight improvement in postoperative functional outcomes [115,116]. In contrast, preliminary data on spinal surgery suggest a potential reduction in blood loss and length of hospital stay in these patients.

### 3.4. Predictive Analysis and Precise Stratification

Predictive models in orthopaedics address postoperative complications, readmissions, eligibility for arthroplasty, and patient-reported outcomes, such as satisfaction and return to functional fitness [117,118,119]. The reported area under the curve values for venous thromboembolism (VTE) after endoprosthesis replacement range from approximately 0.71 to 0.982, indicating moderate to excellent discriminatory ability [95,120]. Models predicting the risk of dislocation after hip arthroplasty achieved approximately 95% accuracy, while eligibility models achieved approximately 87.8% accuracy, with AUC values of 0.97–0.98 [76,121]. Despite these promising indicators, most predictive models have been developed and validated internally in single-center cohorts with limited external validation, and virtually no prospective impact studies have been conducted. The conceptual framework for predicting biological responses in regenerative orthopaedics remains in the hypothesis-generation phase.

### 3.5. Rehabilitation Intelligence and Teleorthopaedics

The applications of AI in rehabilitation [14,41,122] and teleorthopaedics demonstrate that orthopedic care can extend beyond hospitals to patients’ homes and communities [123]. Computer vision systems applied to video recordings can quantify gait asymmetry and joint range of motion [124]. Platforms based on wearable sensors and AI algorithms are being explored for adaptive rehabilitation planning, adherence monitoring, and early detection of suboptimal recovery [125,126,127]. However, the current evidence is limited, largely due to the small number of pilot studies with limited links to long-term outcomes. Teleconsultation platforms that integrate simple AI-based tools to measure range of motion or medical segregation may improve access to care in underserved regions but raise concerns about digital exclusion, data security, and clinical accountability for remotely supervised programs [128,129]. The feasibility of thermal imaging applications is still in its early stages of development.

### 3.6. System-Level Governance, Ethics, and Future Trajectories

In addition to individual applications, the literature highlights significant system-level barriers related to data quality, interoperability, regulatory oversight, and clinician trust. Many orthopedic AI systems have been developed using data from a single center or homogeneous datasets, which limits their overall utility and may exacerbate bias if underrepresented populations are not adequately included [130,131]. The lack of explainability and the “black box” nature of algorithms often constitute significant barriers to their acceptance in high-risk decision-making environments. In addition, the implementation of such solutions is hampered by fragmented clinical workflows, changing regulations on software as a medical device, and insufficient systematic postmarket surveillance. This highlights the need for multicenter training datasets, methods that are easy to explain to clinicians, implementation of science research, and long-term concepts, such as learning teleorthopedic systems and “digital twins” of patients [12,132]. Table 1 summarizes the study types, primary AI applications, and anatomical regions of the 40 included studies.

Systematic reviews were included for contextual synthesis, and primary quantitative extraction focused on original clinical studies.

A summary of diagnostic AI applications in orthopaedics is presented in Table 2.

AI-assisted-surgical planning and intraoperative augmentation are shown in Table 3.

The predictive analytics and precision stratification in orthopaedics are presented in Table 4.

AI-enabled rehabilitation and teleorthopaedics are listed in Table 5.

Table 6 presents the system-level, governance, and implementation frameworks for orthopaedic AI.

The future research agenda derived from expert synthesis is presented in Table 7 below.

Table 8 presents a comparative analysis of outcomes from conventional image interpretation and those enabled by AI support. The data in Table 8 indicate that AI contributes to enhanced performance, improved accuracy, training standardization, and expedited workflows. Nevertheless, the implementation of AI remains dependent on its validation and integration. The reported time metrics correspond to values observed under study-specific or experimental conditions and should not be misconstrued as representing the total duration of clinical workflows.

It is essential to differentiate between the inference time and the entire clinical workflow duration, which includes image transfer, result evaluation, management of false positives, and subsequent clinical decision-making.

## 4. Discussion

### 4.1. Key Findings and Maturity of Evidence

**Figure 2:**
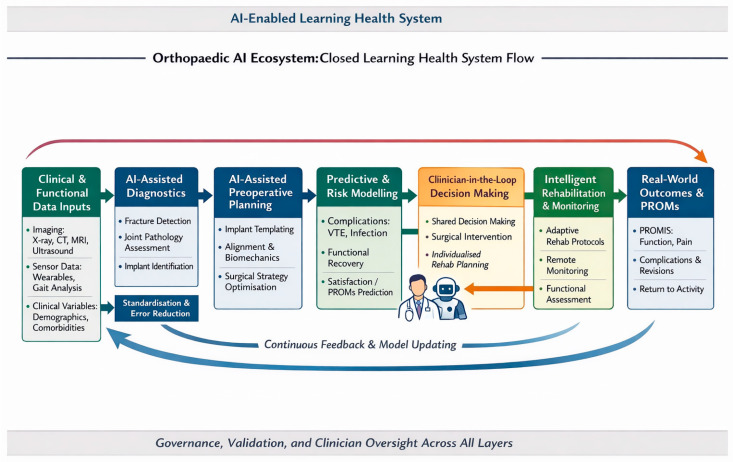
Orthopaedic AI ecosystem flowchart. The conceptual flowchart depicts how imaging data, clinical variables, PROMs, and sensor-derived signals feed AI-assisted diagnostics, arthroplasty planning, outcome prediction, and rehabilitation monitoring, with real-world outcomes fed back into model updates, thereby forming a learning health system for musculoskeletal care.

The key message of this review is that AI in orthopaedics can provide the greatest clinical value when applied to well-defined, standardized tasks with clear validation pathways, thereby augmenting physicians [156]. By contrast, performance and reliability decline as clinical complexity, contextual variability, and the diffusion of responsibility across care processes increase in the healthcare system. This narrative review indicates that AI in orthopaedics is the most clinically mature in diagnostic imaging [8,45,52,98,131,157,158,159,160,161] and arthroplasty planning [11,35,74,115,162,163], where deep learning systems consistently achieve expert-level performance in fracture detection [25,50,52,88,159,160,164,165,166], implant identification [73,167,168,169,170,171], and component template creation [29,30,31]. AI can reduce diagnostic delays [161], planning errors [147,161,169], and perioperative burdens [130]. In contrast, predictive analytics [93,112,172,173], rehabilitation intelligence [116,122,174,175,176], and digital ecosystem concepts (Figure 2), such as learning systems [173,177] or digital twins [157,178], remain at an earlier stage of development, supported mainly by proof-of-concept studies with limited multicenter validation or prospective impact data.

### 4.2. Comparison with Previous Works

Previous reviews have typically focused on individual elements of the orthopedic care pathway, such as radiographic analysis [61,127,132,141,142], knee or hip arthroplasty planning [26,91,124,143,144,145], and general machine-learning-based predictive models [179]. In contrast, this review integrates AI applications in diagnostic imaging [8,48,180,181], surgical planning [12,28,35,44,84,182], risk prediction [173,174,183,184,185], rehabilitation [5,14,41,43,181], and system-level management. It explicitly links these to PROM integration [7,14,153], teleorthopaedics [154,186], and new concepts in learning-based healthcare systems, thereby highlighting the maturity of each area. Many previous reviews have focused on the application of AI in various orthopedic subspecialties. In contrast, this synthesis emphasizes the degree of translational readiness, which is influenced more by task structure and validation feasibility than algorithmic complexity.

### 4.3. Technical Challenges in AI Implementation

Despite significant advances, several technical hurdles impede the translation of AI into routine orthopaedic practice. One major issue is overfitting owing to relatively small or biased datasets, underscoring the need for approaches such as federated learning to pool data across institutions without sharing sensitive patient information [156,157]. Additionally, the “black-box” nature of many AI models raises concerns; explainable AI (XAI) techniques (e.g., SHAP value analysis or LIME) are required to interpret model decisions, thereby enhancing clinician trust in AI outputs [187].

### 4.4. Clinical and Healthcare System Implications

For clinicians, the most immediate opportunities arise from implementing AI to streamline existing processes in radiology and arthroplasty planning [20], where tools can standardize templates [29], prioritize cases, and support less-experienced readers without replacing human judgment.

AI-based teleorthopedic pathways can improve remote medical triage [188,189], range-of-motion assessment [155], and rehabilitation monitoring [41,189,190]. However, implementing these systems requires establishing a robust governance and accountability framework to ensure data security and support digital inclusion [171,172,173], thereby mitigating the risk transferred from institutions to patients and physicians [191,192].

From a healthcare perspective, AI-based risk stratification [148] and PROM-based prediction models [159] may ultimately support personalized thromboprophylaxis [36,37,109], joint decision-making regarding arthroplasty candidates [20,193,194], and more efficient resource allocation. In contrast, teleorthopedic and rehabilitation applications remain constrained by limited validation, contextual variability, and unresolved questions regarding digital equity, data security, and clinical responsibility in remote care models [161,179]. The results emphasize that the effective use of AI in orthopaedics depends not only on the sophistication of the algorithms themselves, but also on clearly defined tasks, robust validation strategies, and effective integration of these tools into existing clinical and organizational frameworks. Even more important than the “sophistication” of the models is whether we know exactly what they are supposed to be used for, how reliably we check their performance, and how we fit them into a real-world workflow. Expanding the use of AI beyond narrowly defined tasks without adequate validation and oversight can undermine physician trust in these systems and ultimately harm the quality of care. Although quantitative data pooling (meta-analysis) was infeasible owing to study heterogeneity, we deliberately prioritized the qualitative synthesis of AI model architectures and use cases. This approach provides technical insights that are often missing from clinical reviews.

### 4.5. Economic and Implementation Considerations

Current evidence on the cost-effectiveness of artificial intelligence (AI) in orthopaedics remains limited and highly context-dependent [195,196,197]. Although imaging triage and arthroplasty planning tools have the potential to mitigate delays, reduce rework, and decrease planning variability, the overall economic impact is influenced by the local baseline performance [198,199], case volume, and degree of workflow integration, including PACS/EHR [112], reporting, and liability pathways [79,195]. Initial costs, such as licensing, integration, cybersecurity, and staff training, as well as ongoing maintenance expenses, including monitoring, model updates, and post-market surveillance, are often underreported, thereby constraining comprehensive health technology assessments [26,79,195,200,201,202].

From an institutional standpoint, the business case for AI is generally most compelling when it demonstrably alleviates bottlenecks, such as radiology turnaround time and revision planning time, or standardizes performance for less-experienced practitioners [74,196,201,203,204]. However, financial incentives may be misaligned if cost savings benefit one stakeholder while another incurs implementation costs. Consequently, future evaluations should incorporate economic endpoints alongside clinical outcomes, encompassing resource utilization, time-to-decision, complication-related costs, and patient-reported values [205,206].

### 4.6. Strengths and Limitations of This Review

The structured narrative, broad thematic scope covering the entire spectrum of orthopedic patient care from imaging, surgery planning, prognosis, rehabilitation, and treatment within a single coherent structure, and the adoption of clear performance indicators and clinically relevant endpoints can be considered strengths of our review.

However, this was not a formal systematic review and did not constitute exhaustive or quantitative synthesis. The evidence base remains dominated by retrospective single-center studies based on carefully selected datasets with limited demographic and institutional diversity. The review was based solely on English-language literature and did not consider studies from gray literature or other languages. Consequently, residual selection and classification biases cannot be excluded. The impact of incomplete consideration of relevant evidence, particularly in areas with heterogeneous imaging protocols, variable annotation standards, and limited external validation, cannot be ruled out.

Several additional limitations of this study should be considered when interpreting these results. Studies vary considerably in terms of the input data, reference standards, outcome definitions, and reporting practices. Image acquisition parameters, real-world annotation strategies, and performance metrics have not been analyzed in sufficient detail, making it difficult to compare models and meaningfully aggregate results. In many areas, the reported accuracy likely reflects optimization for specific tasks rather than the actual clinical performance. Few studies have included rigorous external validation, prospective evaluation, or direct comparison with established clinical processes. It can also be assumed that high algorithm performance does not necessarily translate into better patient outcomes, greater efficiency, or better decision quality. This gap is particularly evident in predictive analytics, rehabilitation intelligence, and teleorthopaedic applications, where long-term outcomes and contextual factors are critical. In addition, many studies have provided limited information on transparency, explainability, fairness, and other ethical issues. Despite growing regulatory and ethical expectations, there is a lack of systematic assessment of bias, interpretability, and failure modes, making it difficult to predict unintended consequences and potentially undermining clinicians’ trust in AI. This may be particularly relevant when AI systems are deployed outside of narrowly defined use cases. Overall, the current literature reflects an evolving, methodologically heterogeneous field, and the reported results should be interpreted with caution when extrapolating to routine clinical practice.

Another concern is the apparent optimism in many early-stage studies, often authored by the same teams that developed the evaluated algorithms. Although these studies often demonstrate high performance under controlled conditions, they may be biased by optimism resulting from the favorable selection of datasets, task formulation, or evaluation protocols tailored to the strengths of the proposed methods. Consequently, the actual performance in heterogeneous clinical settings with unknown data distribution and daily workflow constraints may be significantly lower than previously reported. This highlights the need for independent validation, external replication, and prospective evaluation to distinguish true clinical utility from proof-of-concept results and to avoid overestimating the capabilities of current AI systems that can be safely delivered in practice.

### 4.7. Generative AI

Emerging Generative AI tools warrant consideration in orthopaedics. Although current applications are dominated by discriminative models (for classification or segmentation), generative approaches, including large language models (LLMs), are beginning to assist with clinical documentation and decision support. For example [207], preliminary studies have shown that LLMs can draft discharge summaries and operative reports significantly faster than clinicians, while maintaining comparable quality. These models could reduce administrative burdens and improve patient communication, although issues such as content ‘hallucinations’ and the need for rigorous validation currently limit their clinical deployment [208].

### 4.8. Directions for the Future

Future research should prioritize the prospective multicenter validation of imaging and preoperative planning algorithms. However, the current body of evidence has been limited by insufficient external validation. Therefore, future studies should employ pragmatic designs, such as stepped-wedge cluster-randomized trials or multicenter prospective cohort studies, to assess the real-world clinical impact of routine workflows.

The evaluation should extend beyond the diagnostic accuracy. Key outcomes include patient-reported outcome measures, complications and safety endpoints, time-to-decision, workflow efficiency, downstream healthcare utilization (e.g., additional imaging or re-consultations), and economic outcomes relevant to hospitals and payers.

Validation should not rely solely on a single test. Regular local reevaluation at individual centers is necessary to account for dataset shifts, evolving practice patterns, and changes in the patient population. Research should transition from isolated case reports to implementation-focused studies that assess the impact of AI on treatment outcomes, clinician behavior, and resource utilization.

In the fields of rehabilitation and teleorthopedics, robust prospective studies are required to determine whether remote monitoring and AI-supported adaptive therapy can enhance clinical outcomes and improve patient safety. At the health-system level, future work should address regulatory adaptation, transparent and interpretable model design, continuous post-market oversight, and the integration of patient-reported outcomes and real-world evidence into learning health systems to ensure the safe, ethical, and equitable deployment of AI technologies.

At the system level, it is critical to adapt to changing regulations; develop transparent and understandable models; conduct ongoing oversight after innovations are introduced to the market; and integrate patient-reported outcomes (PROMs) and real-world evidence into health education systems to ensure the safe, ethical, and equitable implementation of these solutions.

## 5. Conclusions

AI in orthopaedics and in the care of patients with musculoskeletal disorders has reached clinical maturity in terms of diagnostic imaging and arthroplasty planning. In these areas, algorithms can consistently achieve expert-level results, leading to measurable improvements in efficiency and perioperative decision-making. In contrast, areas such as predictive analytics, rehabilitation intelligence, and teleorthopaedic applications are still at an early stage of translational readiness. However, their development is hampered by limited external validation, biased or narrowly representative datasets, and a lack of transparency in algorithmic decision-making processes. The transition from proof-of-concept implementations to reliable clinical services will rely, to some extent, on further improvements in the algorithm performance. However, to a large extent, implementation will require robust multicenter prospective evaluations, repeatable local validations, systematic assessments of fairness and explainability, and clinically meaningful integration of PROM and sensor data. Under these conditions, AI is well-positioned to complement, rather than replace, orthopedic knowledge, thereby supporting more consistent, patient-centered, and value-based musculoskeletal care.

## Figures and Tables

**Table 1:** Characteristics of the included studies on AI in orthopaedics.

Study	Was the Full Text Used for Data Extraction?	Study Type	Primary AI Application	Anatomical Region
D. Langerhuizen et al., 2019 [50]	Yes	Systematic review	Fracture detection/classification	Ankle, hand, hip, spine, wrist, proximal humerus
Xiang Zhang et al., 2022 [8]	No	Systematic review/meta-analysis	Fracture diagnosis	Multiple (fractures)
J. Karnuta et al., 2023 [71]	No	Validation study	Knee implant identification	Knee
Jakub Olczak et al., 2017 [17]	Yes	Validation study	Fracture detection	Wrist, hand, ankle
Lok Sze Lee et al., 2022 [72]	Yes	Narrative review	OA diagnosis, TKA outcome prediction	Knee
M. Ren et al., 2021 [73]	No	Systematic review	Implant identification	Not specified
Adriaan Lambrechts et al., 2022 [74]	Yes	Development study	TKA preoperative planning	Knee
B. Gurung et al., 2022 [75]	No	Systematic review	THA/TKA image analysis	Hip, knee
P. Hernigou et al., 2022 [76]	No	Systematic review of review	Dislocation risk prediction	Hip
Florent Bernard de Villeneuve et al., 2022 [77]	No	Development study	Lower limb alignment analysis	Lower limb
Aakash K. Shah et al., 2023 [78]	Yes	Systematic review	TJA implant analysis	Hip, knee
Songlin Li et al., 2023 [79]	Yes	Retrospective cohort	TKA preoperative planning/PSI	Knee
Anjali Tiwari et al., 2022 [80]	Yes	Validation study	Knee OA grading	Knee
Xi Chen et al., 2022 [81]	Yes	Prospective cohort	THA preoperative planning	Hip
M. Bonnin et al., 2023 [82]	No	Development/validation	TKA radiographic analysis	Knee
J. Karnuta et al., 2021 [83]	No	Validation study	Knee implant identification	Knee
Di Xue et al., 2025 [11]	No	Systematic review/meta-analysis	THA preoperative planning	Hip
Jichuan Wang et al., 2024 [84]	Yes	Proof-of-concept	Screw reconstruction planning	Pelvis
U. Longo et al., 2025 [85]	Yes	Systematic review	THA outcome prediction	Hip
Nalin Zadoo et al., 2025 [86]	No	Validation study	Pediatric bone age assessment	Hand/wrist
Anna Lind et al., 2021 [87]	Yes	Validation study	Knee fracture classification	Knee
Pengran Liu et al., 2021 [88]	No	Validation study	Tibial plateau fracture diagnosis	Knee
O. Musbahi et al., 2025 [89]	No	Systematic review/meta-analysis	OA classification and prognosis	Not specified
J. Karnuta et al., 2023 [90]	No	Validation study	THA implant identification	Hip
M. Smolle et al., 2022 [91]	Yes	Controlled trial	Knee OA assessment	Knee
Dong Wu et al., 2020 [92]	No	Clinical trial	THA preoperative planning	Hip
Farid Al Zoubi et al., 2022 [93]	No	Cohort study	OR efficiency optimization	Hip, knee
D. Houserman et al., 2022 [94]	No	Validation study	Arthroplasty candidacy prediction	Knee
Davood Dalil et al., 2025 [95]	No	Systematic review	VTE prediction	Hip, knee
Rosmarie Breu et al., 2024 [96]	Yes	Retrospective study	Distal radius fracture detection	Wrist
P. Passias et al., 2022 [97]	No	Retrospective cohort	Spinal deformity surgery	Spine
Rayane Benhenneda et al., 2023 [98]	No	Diagnostic study	Arthroscopic LHB diagnosis	Shoulder
Adeel Anwar et al., 2024 [31]	No	Prospective study	THA preoperative planning	Hip
Xi Chen et al., 2022 [99]	No	Prospective study	THA PSI-assisted surgery	Hip
Qing Lan et al., 2024 [30]	Yes	Retrospective cohort	TKA preoperative planning	Knee
Nikolas J. Wilhelm et al., 2024 [100]	No	Multicenter validation	Lower extremity alignment	Knee
Michael P. Murphy et al., 2023 [101]	Yes	Retrospective cohort	Cup orientation measurement	Hip
Phichai Udombuathong et al., 2022 [102]	Yes	Retrospective diagnostic	Hip fracture diagnosis	Hip
Gang Zhang et al., 2024 [103]	No	RCT	THA preoperative planning	Hip
Bingshi Zhang et al., 2023 [104]	No	Retrospective cohort	THA preoperative planning	Hip

“Yes” indicates studies from which quantitative performance data or outcome measures were extracted. “No” indicates studies included for contextual or conceptual synthesis only (e.g., systematic reviews or narrative reviews).

**Table 2:** Evidence summary of AI applications in orthopedic diagnosis.

Application Domain	Model Types	Reported Performance	Clinical Benefit	Key Limitations
Fracture detection	CNNs (ResNet, DenseNet, VGG), transfer learning	Sensitivity 90%, specificity 92%	Reduced diagnostic error; fast execution (<1 s/image)	Mostly single-centre training; limited external validation
Distal radius fracture analysis	Deep learning radiograph interpreters	↑ Sensitivity 80→87%; ↓ error 14→9%	Performance boost for non-experts	Trust and liability concerns; data heterogeneity
Implant identification	CNN classifiers & radiograph feature extractors	Accuracy 97–99%	Faster revision planning; component sourcing	Limited integration into PACS/theatre workflow
Osteoarthritis grading (KL, MRI)	CNNs/ensemble learning	Accuracy up to 93%; improved inter-observer agreement	Standardisation, reproducibility	However, the black box” reasoning remains unexplained in this study.
Thermal imaging for inflammation	AI segmentation & pattern recognition	Early feasibility only	Potential remote monitoring tool	Lacks robust clinical validation

Arrow up—increase; arrow down—decrease, arrow right—result.

**Table 3:** Evidence summary of AI in surgical planning and execution.

Surgical Area	Key Functions	Evidence of Benefit	Magnitude of Effect	Constraints
THA planning	Component templating, segmentation, sizing	More accurate sizing vs. manual	Acetabular matching ↑ from 30–57% to 66–90%	AI platform integration into conventional OR planning
THA operative metrics	Pre-op planning support	Improved intraoperative performance	−12 min OR time, −50 mL blood loss, ↓ correction rate by 40%, HHS + 1.42	Lack of prospective trials
TKA templating	Femoral & tibial sizing	Higher accuracy vs. manual	92.9% vs. ~45%	Variability between centres
Segmentation/PSI creation	Accelerated 3D workflow	Reduced processing time	Segmentation 129 → 4 min; PSI planning 160 → 35 min	Workflow dependency on vendor systems
Spine deformity surgery	Intraoperative decision support	Fewer complications and shorter stay	↓ blood loss (*p* = 0.001), ↓ LOS (*p* = 0.012)	No standard regulatory pathway
Surgical navigation	Automated positioning and orientation calculations.	Real-time validation	Cup angle calculation in 0.22 s	Explainability gaps

Arrow up—increase; arrow down—decrease, arrow right—result.

**Table 4:** Predictive AI models and clinical outcome decision support.

Predictive Target	Performance Reported	Clinical Utility	Validation Maturity
VTE risk after arthroplasty	AUC 0.71–0.982	Tailored prophylaxis evaluation	Limited external validation
THA dislocation risk	Accuracy 95%	Component alignment strategy	Minimal generalisability studies
Arthroplasty candidacy prediction	Accuracy 87.8%; AUC 0.97–0.98	Supports shared decision-making	No prospective testing
Satisfaction/recovery modeling	Feasibility demonstrated	Early expectation management	Feature attribution unclear
Biologic responder prediction (conceptual)	Theoretical modeling	Precision orthobiologics	No validated clinical tool

**Table 5:** AI in rehabilitation, monitoring, and teleorthopaedics.

Function	Use Case	Evidence Strength	Benefits	Barriers
Computer vision gait analysis	Asymmetry detection, movement scoring	Moderately strong feasibility	Remote access, reproducibility	No RCT linking to outcomes
Adaptive rehab planning	Wearables + AI coaching	Emerging	Personalisation + early detection	Adherence variability
Remote triage/ROM assessment	Teleconsultation screening	Early clinical deployment	Improved access	Digital divide
Thermal imaging monitoring	Diabetic foot, inflammation	Conceptual translation	Non-invasive early alerts	Needs validation trials

**Table 6:** Systems, Ethics, and Regulatory Landscape for AI in Orthopaedics.

Issue	Evidence Described	Implication	Research Need
Explainability gap	A barrier to surgeon trust	Slows adoption	XAI models for planning + diagnostics
Data heterogeneity	Single-centre datasets	Poor generalisability	Multicentre training datasets
Workflow fragmentation	Lack of PACS/robotics interoperability	Translation gap	Implementation science
Algorithmic bias	Risk of unequal access	Ethical hazard	Bias detection frameworks
Undefined liability	No clear accountability	Legal risk	Regulatory standards

**Table 7:** Future research agenda derived from expert synthesis.

Priority Area	Rationale	Target Outcome
Digital twins	Surgery simulation before intervention	Individualised pre-trial planning
PROMIS–AI fusion	Underused dataset for modeling	Phenotype-driven rehab + decision aids
Precision orthobiologics	Variable outcomes of PRP/IAHA	Algorithmic responder identification
Teleorthopaedics learning loops	Continuous monitoring data streams	Real-time model updating
Fair-AI governance	Safety > accuracy	Bias-safe clinical deployment

**Table 8:** Comparative performance: AI vs. traditional clinical workflows.

Domain	Conventional	AI-Enhanced	Observed Gain
Fracture detection	0.5–3.0 min (average 1.2–1.5 min)/X-ray [133,134]	0.5–2.5 min (average 1.0–1.3 min)/X-ray [135,136]	Significant efficiency boost with reduced missed fractures. Improved sensitivity, 29% fewer missed fractures, and optimized working time through AI-supported prioritization rather than raw interpretation speed alone.
Distal radius interpretation accuracy	80% sensitivity; 14% error [137,138]	87% sensitivity; 9% error [139,140]	↑ precision, especially for non-expert readers. Improved sensitivity and fewer missed fractures, particularly with segmentation-based AI support.
Implant identification	~60–80% accuracy; time-consuming, and operator-dependent [141]	~96–99% accuracy (manufacturer + model), AUROC ≈ 0.99 [142]	Rapid revision planning; reduced uncertainty
Arthroplasty templating accuracy	~40–60% exact size match [143,144]	~70–90% exact size match; ~3–4× higher odds of correct sizing vs. manual 2D [143,145]	Improved implant size prediction and positioning, enabling better THA planning and potentially shorter operative time.
CT segmentation for TKA/PSI	129 min processing [79,146]	4 min with AI [79,146]	Reduction in CT processing time (≈20–30×), enabling rapid 3D planning and PSI generation without compromising segmentation accuracy. Processing time refers to computational segmentation time and does not represent total clinical workflow duration
PSI planning time	160 min [147]	35 min [147]	Improved scheduling, accelerated PSI planning and prototyping (substantially shortened planning time) while maintaining sub-millimetric accuracy of cutting guides.
Spinal surgery complication reduction	Standard complication risk and length of stay (e.g., transfusions ~28%, LOS ~5.1 days in short lumbar fusions) [148]	↓ complications with AI (*p* = 0.021) lower rate of selected complications (e.g., transfusions 23% vs. 28%) and shorter hospital stays (4.8 vs. 5.1 days), ↑ accuracy in screw placement and fewer reoperations [149,150]	Lower complication rates and shorter stay with AI-supported navigation and risk prediction, enhancing safety in spine surgery
Teleorthopaedic triage	In-person review required; manual ROM goniometry; limited reach [151,152,153,154]	Remote screening + ROM estimation [155]	Expanded access, triage efficiency, and early feasibility evidence

Arrow up—increase; arrow down—decrease.

## Data Availability

Data supporting the reported results are available from the authors upon request.

## Supplementary Materials

The following supporting information can be downloaded at: https://www.mdpi.com/article/10.3390/jcm15051751/s1, Table S1: Full-text articles excluded after eligibility assessment, and reasons for exclusion; Table S2: Qualitative summary of methodological quality of included studies.
