# Supplementary material for: Artificial Intelligence in Orthopaedics: Clinical Performance, Limitations, and Translational Readiness—A Review
Source: J Clin Med. 2026 Feb 25;15(5):1751. doi: 10.3390/jcm15051751 (PMC12985454; doi:10.3390/jcm15051751)
Supplement: Supplementary file 1 [file jcm-15-01751-s001.zip › jcm-4166651-Supplementary Table S1.pdf]

Supplementary Table S1. Full-text articles excluded after eligibility assessment, and reasons for exclusion

Category of exclusion	Number of studies (n)	Representative reasons
No clinical validation	18	Algorithm development only; simulation-based evaluation without clinical data
Engineering-focused without a clinical framework	12	Image processing or model optimisation studies lacking clinical endpoints
Robotics or navigation systems without decision-support relevance	8	Technical navigation accuracy only; no impact on clinical decision making
Duplicate cohorts or overlapping datasets	7	Same or substantially overlapping patient populations reported elsewhere
Conference abstracts, editorials, or narrative commentaries	9	No peer-reviewed full-text data available
Non-English publications	4	Outside predefined language inclusion criteria
Other (insufficient outcome reporting)	2	Lack of quantitative performance metrics or translational relevance
Total excluded	60	
