# Supplementary material for: Artificial Intelligence in Orthopaedics: Clinical Performance, Limitations, and Translational Readiness—A Review
Source: J Clin Med. 2026 Feb 25;15(5):1751. doi: 10.3390/jcm15051751 (PMC12985454; doi:10.3390/jcm15051751)
Supplement: Supplementary file 1 [file jcm-15-01751-s001.zip › jcm-4166651-Supplementary Table S2.pdf]

Supplementary Table S2. Qualitative summary of methodological quality of included studies

Methodological domain	Predominant pattern across included studies
Study design	Predominantly retrospective, single-centre observational studies
Sample size	Small to moderate cohorts; limited statistical power in several studies
Population diversity	Restricted demographic and institutional diversity
Reference standard	Expert consensus or retrospective clinical labels; limited use of longitudinal clinical outcomes
Validation strategy	Internal validation common; external multicentre validation uncommon.
Prospective evaluation	Rare; only a small minority of studies employed prospective or trial-based designs.
Outcome reporting	Focus on technical performance metrics (accuracy, sensitivity, AUC); limited reporting of clinical outcomes or workflow impact.
Bias assessment	Incomplete reporting of selection bias, dataset shift, and failure modes
Model transparency	Limited reporting of explainability, calibration, and uncertainty estimation
Clinical integration	Evaluation often performed outside routine clinical workflows.
